# Dynamic modulation of the Fermi energy in suspended graphene backgated devices

**DOI:** 10.1080/14686996.2019.1612710

**Published:** 2019-06-03

**Authors:** Omar M. Dawood, Rakesh Kumar Gupta, Umberto Monteverde, Faisal H. Alqahtani, Hong-Yeol Kim, James Sexton, Robert J. Young, Mohamed Missous, Max A. Migliorato

**Affiliations:** a School of Electrical and Electronic Engineering, University of Manchester, Manchester, UK; b School of Materials, University of Manchester, Manchester, UK; c Department of Physics, College of Education for Pure Science, University of Anbar, Anbar, Iraq; d Manchester Institute of Biotechnology, University of Manchester, Manchester, UK; e Physics Department, Faculty of Science, King Khalid University, Abha, Saudi Arabia; f National Graphene Institute, University of Manchester, Manchester, UK

**Keywords:** Suspended graphene, Raman spectroscopy, atomic force microscopy, 10 Engineering and Structural materials, 104 Carbon and related materials, 201 Electronics / Semiconductor / TCOs, 208 Sensors and actuators, 503 TEM, STEM, SEM, 505 Optical / Molecular spectroscopy

## Abstract

Freestanding (suspended) graphene films, with high electron mobility (up to ~200,000 cm^2^V^−1^s^−1^), good mechanical and electronic properties, could resolve many of the current issues that are hampering the upscaling of graphene technology. Thus far, attempts at reliably fabricating suspended graphene devices comprising metal contacts, have often been hampered by difficulties in exceeding sizes of 1 µm in diameter, if using UV lithography. In this work, area of suspended graphene large enough to be utilized in microelectronic devices, have been obtained by suspending a CVD graphene film over cavities, with top contacts defined through UV lithography with both wet and dry etching. An area of up to 160 µm^2^ can be fabricated as backgated devices. The suspended areas exhibit rippling of the surfaces which simultaneously introduces both tensile and compressive strain on the graphene film. Finally, the variations of the Fermi level in the suspended graphene areas can be modulated by applying a potential difference between the top contacts and the backgate. Having achieved large area suspended graphene, in a manner compatible with CMOS fabrication processes, together with enabling the modulation of the Fermi level, are substantial steps forward in demonstrating the potential of suspended graphene-based electronic devices and sensors.

## Graphical Abstract

**Figure:**
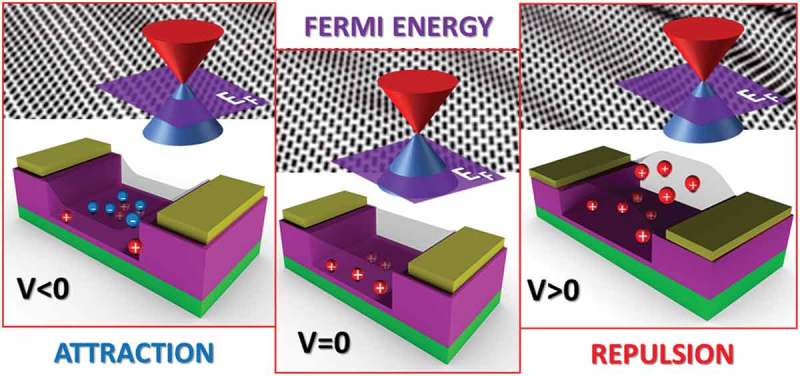

## Introduction

Since its first isolation, graphene [1] has been hailed as a revolutionary material. However, in order to enable large volume applications in the electronic and sensors sectors, graphene still needs to be produced as a large transferrable film [2–4]. Industrial wafer-scale fabrication has encountered upscaling challenges, as large area uniformity of graphene grown by chemical vapor deposition (CVD), even before any wet chemical transfer, is still not sufficiently quality assured. This is why obtaining defect-free CVD graphene, as crystalline as possible (e.g. grain sizes larger than 100 µm in diameter), has seen increased interest in recent years [5–7]. Furthermore, the intrinsic properties of pristine graphene are severely affected by both its interaction with the host substrate [8,9] and charge trapping (or contamination) that originates from both the conventional wet chemical transfer of the CVD graphene-polymer stack [10–12], and any subsequent processing typically involving the use of polymeric photoresist [13–15]. Current-induced or laser ablation cleaning of freestanding (suspended) graphene films can resolve many issues [16–20]. However, attempts at suspending graphene within a microelectronic device layout, have found that the largest suspensions possible are often limited in size, with at least one direction not exceeding 1 µm. Even then a number of studies reported better mechanical, electronic and chemical properties of suspended graphene (SusG) compared to supported graphene [16,21,22] (SupG), e.g. electron mobility increased up to ~200,000 cm^2^V^−1^s^−1^ (from 2000 to 20,000 cm^2^V^−1^s^−1^ for SupG) [16]. The increased mobility of SusG can be explained with the removal of the van der Waals interaction between graphene and the surface atoms of the substrate. The increased conductivity (the product of mobility and the carrier density) is instead linked to the presence of naturally forming ripples in freestanding graphene [23] resulting in higher Fermi energies in the conduction band, creating an excess of electrons.

In this paper, a combined atomic force microscopy (AFM) and Raman spectroscopy (RS) approach was used to investigate the nature of backgated devices comprising SusG, in terms of both the in-built static strain inherently present and the additional dynamic strain that can be produced by applying a bias to the electrodes.

## Device fabrication

Four different devices were fabricated. For all samples, CVD graphene was used. The four devices mainly differ in the nature of the insulating material used to define the cavities, the shape and dimensions of the cavities, and the (semi)conductor used to form the backgate electrode. The four devices are as follows: #1, SU-8 with rectangular cavities (15 × 8 µm^2^) and aluminium backgate; #2, SU-8 with rectangular cavities (20 × 8 µm^2^) and aluminium backgate; #3, SU-8 with circular cavities (5 × 5 µm^2^) with indium tin oxide (ITO) backgate; #4, SiO_2_ with multiple square cavities (5 µm diameter) and n-type silicon backgate.

For devices #1 and #2, a thin film of aluminum with a thickness of 200 nm was evaporated on the Si surface to provide a backgate contact. A SiO_2_ layer of 300 nm thick was then created over the aluminum, using radio frequency (RF) sputtering. After that, a 2.5 µm SU-8 (a negative photoresist) was spin-coated over the silicon dioxide layer. Next, reactive ion etching (RIE) was used to define the cavities. For device #3, the same process used for device #1 and #2 was repeated, except now an ITO layer with a thickness of 200 nm was grown on the Si surface instead of the aluminum layer. For device #4, a similar lithographic process as for devices #1 and #2 was followed but with a SiO_2_ layer of 1.3 µm thickness thermally grown directly on the Si surface.

The detailed process of fabrication of the cavities varies depending on whether these are fabricated in SU8 or SiO_2_:

### Cavities in SU8, samples #1 to #3

SU8 was spin coated onto the Si substrate, to a ~1 μm thick layer, then flood exposed in UV (λ = 365 nm) for 2 min and hard baked at 180°C for ~25 min. A 50 nm thick evaporated Al hard mask patterned with cavities was used to expose the 0.5 μm photoresist (PR) film. Solution development of the top PR (S1805) was followed by the wet-etching of the exposed Al in phosphoric nitric acetic acid (H_3_PO_4_: HNO_3_: CH_3_COOH: H_2_O = 73%: 3.1%: 3.3%: 20.6%) solution. The ~1 μm deep cavities were etched in SU8 by reactive ion etching (RIE, O_2_ gas, at 50-W RF (13.56 mHz) power and gas flow rate of 50 sccm O_2_ and at an etching rate of ~50 nm/min). The Al hard mask was later removed by dipping the substrates in Al etchant solution.

### Cavities in SiO_2_, sample #4

A 150 nm thick Al hard mask layer was evaporated directly on ~1.5 μm thick SiO_2_ layer, grown through wet oxidation of silicon substrate, and lithographically patterned for cavities. Cavities were etched in SiO_2_ by RIE (CF_4_ gas, at 100-W RF (13.56 mHz) power and gas pressure of 22 mtorr and an etch rate of 25 ± 2 nm/minute was achieved to create ~1 μm deep cavities in ~35 ±4 min). The remaining Al hard mask was then removed by Al etchant.

For all devices, CVD graphene was supplied by 2-DTech Ltd, grown on a 25 µm copper film. Graphene was covered by a sacrificial polymer layer and then separated from the copper by wet etching in ammonium persulfate (APS) until all the copper was etched away. The film was then transferred onto a pre-patterned silicon substrate.

Top contacts were thermally evaporated as a thin film of gold (100 nm) using UV lithography. Even though the sacrificial layers were removed from the surface of the exposed graphene, the device still exhibited residual polymer contamination that to be entirely removed, required further steps. For devices #1 to #3, a large part of the removal of the protective polymer layer was done by exposing the surface to acetone in vapor form (acetone fog). This was done in order to make the polymer removal procedure more delicate towards the membranes, which tend to otherwise break rather easily. During this early part of the fabrication process, the cleaned device was, annealed at 300°C under N_2_ environment for 5 min to obtain Ohmic contacts.

## SEM/AFM results

**Figure 1.**
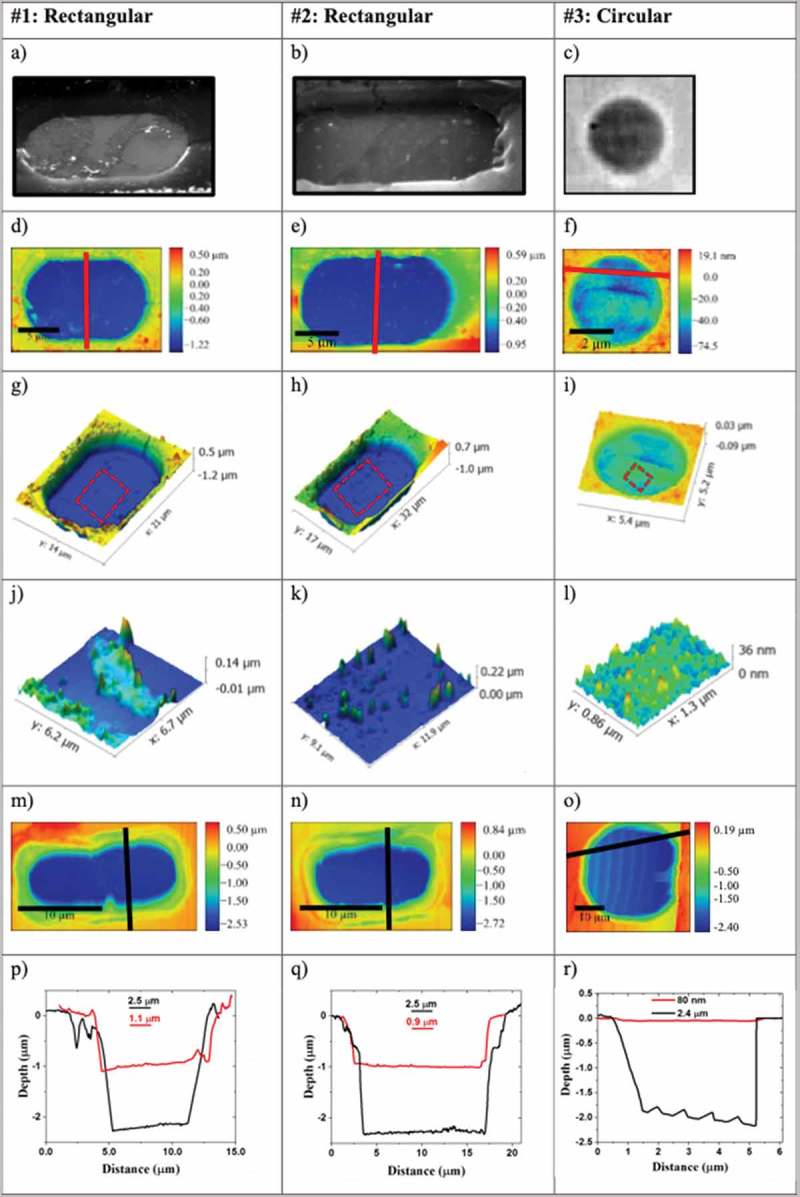
AFM and SEM of three different specimens of suspended graphene, samples #1, #2 and #3, given in the first, second and third columns, respectively. Data in the columns is for sample #1, #2 and #3, respectively. They contain, in sequence for each sample: (a–c) SEM image; (d–f) 2D AFM image; (g–i) 3D counterpart image; (j–l) inset zoomed AFM image taken from the 3D AFM images to test for polymer contamination; (m–o) 2D AFM image of a cavity without graphene; (p–r) height line scans along the red (black lines) indicated in the 2D AFM images of the graphene film (cavity).

Electron microscopy studies of the SusG membranes were performed on samples #1 to #3 (more details are provided in section 1 of the Supplementary Information (SI)). The results are shown in Figure 1, where samples #1 and #2 are rectangular in shape, while sample #3 is circular in shape. Some residual polymer is clearly visible for sample #1, Figure 1(a), while the regular dots evident over sample #2, Figure 1(b), are the result of ablation caused by the laser used during Raman spectroscopy (RS) analysis.

In all three cases, the number of membranes that survived the fabrication process unbroken was limited, often around only 10% or less. Membranes tend to not always be completely broken and the graphene sheet in that case appears to be hanging by a ‘thread’ (see Figure S7 in the SI, section 4). In Figure 1(d–f) we show AFM images of the intact membranes, which have good quality and uniformity. SusG is clearly recessed below the level of the surface as if it has sunk inside the cavity. Figure 1(j) to (l), which are zoomed-in areas (location given in (g–i)), shows evidence of leftover polymer contamination. Though most of the polymer has been removed through the initial cleaning process, areas of contamination remain, which are well known to reduce the electro-optical performance of graphene, albeit providing additional p-type doping [24,25].

Comparison between the AFM data of SusG in Figure 1(d–f) and that of the broken graphene in (m–o), allows an estimate of the amount by which the graphene is suspended relative to the bottom of its cavity, Figure 1(p–r). For samples #1 and #2, the graphene appears recessed by 1.1 µm and 0.9 µm, respectively, while being suspended by 1.4 µm and 1.6 µm over the bottom of the 2.5 µm deep cavity. Sample #3 appears to show no pronounced recession (only 80 nm). This could be due to the fact that more residual polymer appears to be present on the surface (Figure 1(i)) which would make the graphene layer more rigid.

Samples #1 to #3 have rather large areas of SusG: 120 µm^2^, 160 µm^2^ and 25 µm^2^. The SusG being recessed is a result of both gravity and adhesion, most likely through van der Waals interaction, to the side wall of the cavity. Such adhesion is one of the origins of strain over the SusG surface, as will be shown later.

**Figure 2.**
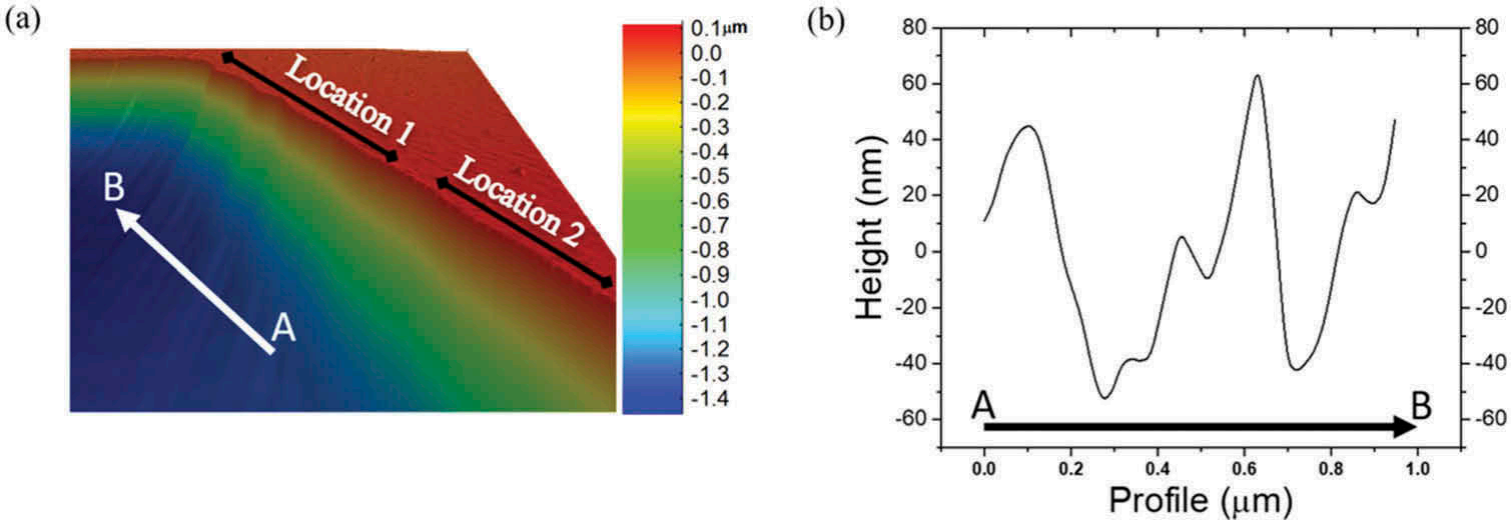
AFM micrograph of suspended graphene, zoomed in the proximity of the cavity. The edges of the cavities are never completely atomically flat: (a) jagged edge (Location 1) is clearly visible as opposed to Location 2; (b) the graphene sheet exhibits a pattern of static ripples where the cavity edges are more jagged.

The edges of the cavities appear, in places, not to be atomically flat, as can be seen in the AFM micrograph in Figure 2(a), where a jagged edge is visible at Location 1. The graphene sheet, which is one atom thick, is very sensitive to any imperfections around the rim of the cavity, and therefore appears to hang from the edges as if pinned in some places but not others Figure 2(a), e.g. pinned at Location 1 more than at Location 2. This creates a pattern of static ripples where the cavity edges are more jagged, as shown in Figure 2(b). Such ripples which will be shown are responsible for local variations in the strain, as also predicted in our previous work [23].

## Raman spectroscopy results

Raman spectroscopy can be used to determine both the doping and strain of the SusG compared to the SupG. Detailed information about the Raman system and parameters used are given in the SI, section 1.

RS is often used to detect the number of graphene layers [26], their crystalline structure, i.e. the presence of defects [27], the concentration of negative or positive dopants [28–31], and the presence/absence of stress [32–34]. Furthermore, the RS polarization measurements can also reveal the presence of different types of stress [35–37]. Guidelines on how to interpret RS data of a typical graphene specimen have been extensively reported [38–41].

**Figure 3.**
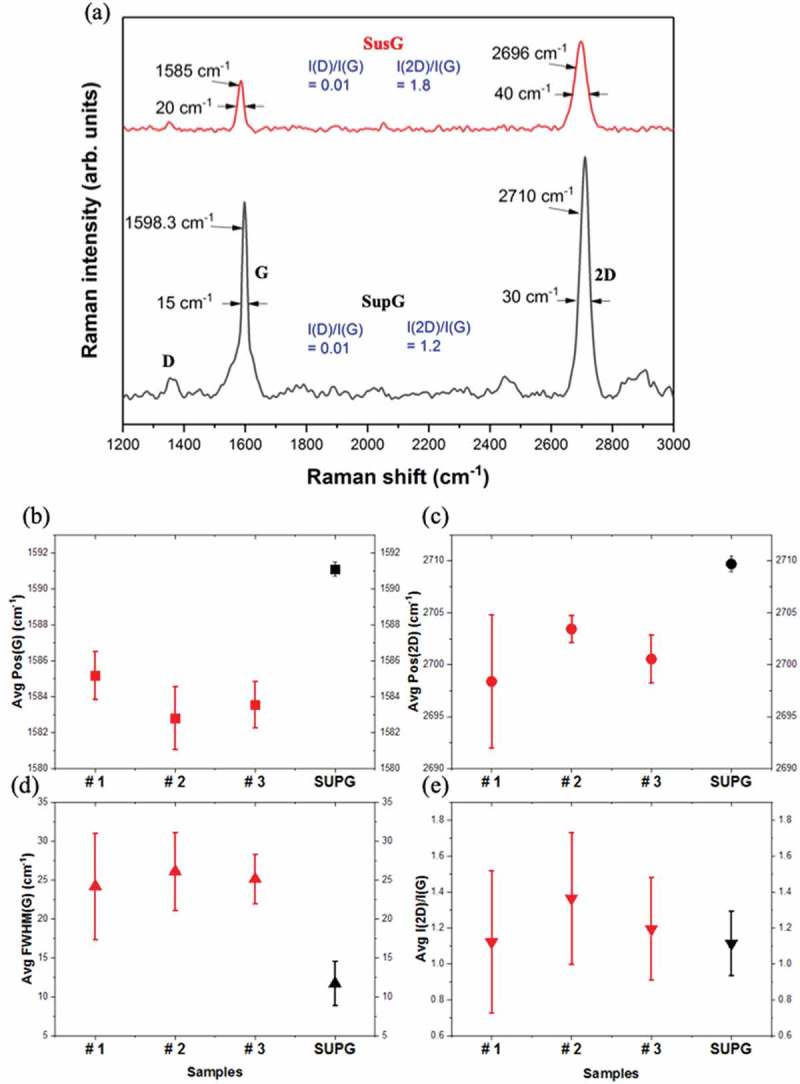
Raman spectroscopy data for three specimens of suspended and one of supported graphene. The Raman spectra were first mapped over several points (10 points, in steps of 500 nm) across the surface of the membranes, and then averaged. (a) Comparison of Raman spectra of SusG (red) and SupG (black), (b) G mode, (c) 2D mode, (d) FWHM of G mode and (e) intensity of 2D to G mode ratio.

Figure 3(a) shows a comparison of typical Raman peaks observed in the SusG and SupG used in this work. The following is a brief guide to interpreting the data: red-shifted G and 2D modes, together with increased full width at half maximum (FWHM), reveal that graphene is under some form of stress [33,42–44]; a high I(2D)/I(G) ratio is evidence of low doping levels [45–47]; the I(D)/I(G) ratio gives an indication of the degree of perfection (in terms of defects and monolayer quality) of the graphene film [48].

Figure 3(b–e) present a comparison between the average RS data taken at different locations over the surface of SusG of samples #1 to #3, and data from areas of SupG. The I(2D)/I(G) ratio (Figure 3(e)) is between 1 and 1.5 across the various points measured and no particular difference can be observed between the SusG and SupG, indicating that the quality and uniformity of the graphene is similar in both areas.

The average peak positions of the G and 2D mode in Figure 3(b,c) show that there is a consistent redshift of around 2 cm^−1^ to 10 cm^−1^, compared to the values recorded for the SupG. Furthermore, there is a much larger spread of values for SusG compared to SupG. This would suggest a significant degree of local variations in either the film uniformity, strain status or doping levels over the surface of each individual SusG area.

Differences between the G and 2D modes of SusG and SupG can help identify the type and level of doping present. For example, p-doping doping could originate from residual photoresist (PR) (in SusG) and n-doping could be due to SU-8 (in SupG) [49]. On the other hand, FWHM analysis of the G band for SusG (red) & SupG (black) (Figure 3(d)) shows that samples #1 and #2 have a larger average FWHM compared to SupG, which clearly indicates that either strain is present in the SusG regions or the doping levels are lower in SusG than of those of SupG. This is an interesting difference, suggestive of the fact that SusG is not heavily recessed into the cavity, and hence results in less strain than for samples #1 and #2. However, the FWHM of the G mode is similar for all the samples measured suggesting the opposite instead, e.g. that strain is similar in all SusG samples, irrespective of their sizes, but yet different from SupG. We, therefore, conclude that the smaller size of the SusG area results in sample #3 exhibiting properties intermediate between SupG and completely freestanding SusG.

Before we move onto the next section, it is worth pointing out that the large spread of peak positions for the 2D mode of sample # 1, can be explained by the presence of anisotropic strain. The 2D mode is activated by a second-order double resonance process near the Dirac point of the band structure of graphene. The presence of any external influence, such as strain, naturally leads to shifs in the electron momentum around the Dirac point, which directly affects the mechanism of generating the 2D mode. By looking carefully at the microscopy images of samples #1 and #2, in Figure 1(a,b), it is apparent that the surface of sample #1 exhibits slightly more residual polymer than sample#2, which would likely result in the presence of higher non-uniform (anisotropic) local strain. A wider distribution of the 2D peak positions for sample #1 compared to sample #2 is therefore expected.

## Modulation of Fermi level in suspended graphene

In graphene, the doping concentrations of electrons and holes, $n_{e,h}$ [50], depend directly on the change in Fermi level (E_F_) [51]. The following equations, from ref [50,51], define E_F_ and doping levels ($n_{e,h}$) as:

(1) $$\Delta E_{F}=D_{ep}*(\Delta Pos\left(G\right)-Pos{\left(G\right)}^{0})$$

(2) $$n_{e,h}=\frac{\Delta {E_{F}}^{2}}{\pi \hbar^{2}v_{F}^{2}}$$

where ℏ is the reduced Planck constant, 6.58,212 ×10^−16^ eV s, *ν_F_* is the Fermi velocity, 10^8^ cm/s [52], $D_{ep}$ is the electron-phonon coupling strength, which has been reported to have values ranging from 18 to 65 meV [53], depending on three parameters: (i) the dielectric constant of a material that surrounds graphene (air or polymer); (ii) the wavelength of the laser used; (iii) the average I(2D)/I(G) ratio at which the Fermi level is close to zero eV, i.e. the highest value of I(2D)/I(G) ratio [46,47]. Taking these factors into account for our study of suspended graphene, the average value of $D_{ep}$ can be taken as 33 meV [53].

From the measured data, it is apparent that the average G and 2D peak positions, for unstrained/undoped graphene can be estimated as $Pos{\left(G\right)}^{0}$ = 1588 cm^−1^ and $Pos{\left(2D\right)}^{0}$ is 2700 cm^−1^. While the former is needed in Equation 1, the latter will only be needed later when determining the strain.

**Figure 4.**
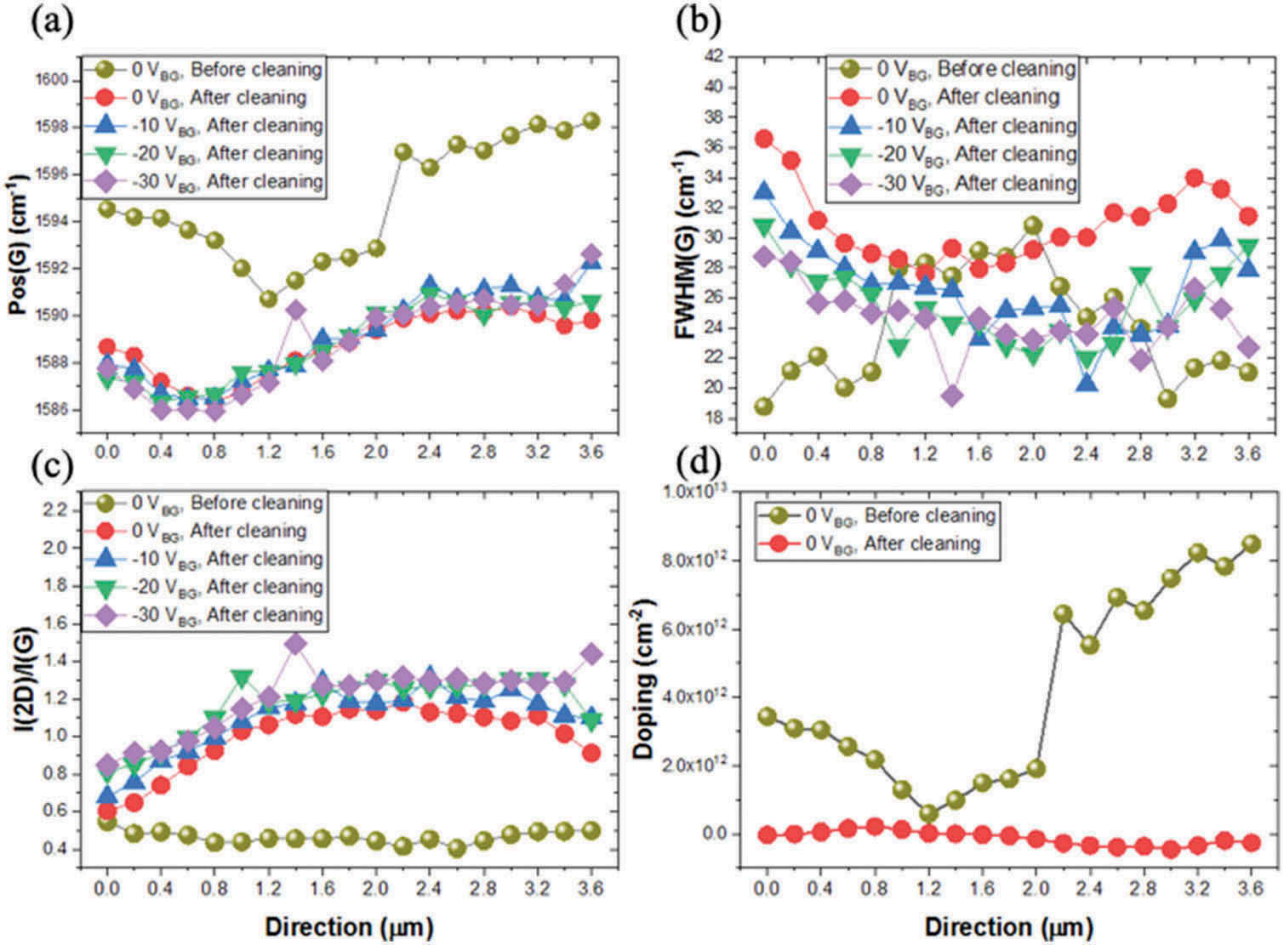
Raman spectroscopy data for sample #2 as a function of V_BG_, before and after the PR is completely removed. The Raman spectra were then mapped over several points (16 points, in steps of 200 nm) across the surface of the suspended membrane. (a) G mode, (b) FWHM of the G mode, (c) intensity of the 2D to G mode ratio and (d) doping levels.

Sample #2 was measured before and after the PR was completely removed. A Raman line mapping (line length ~3.8 µm) was obtained, where Raman data was collected from 19 data points (200 nm apart) along the SusG surface. Figure 4 shows the Raman data (a-c), together with the determined doping levels (d), before cleaning and after cleaning at different backgate biasing voltages (V). From Figure 4, though there are variations in the Raman data, it is confirmed that monolayer graphene is suspended and that the doping concentration obtained from Equations 1–2 is high before cleaning the sample, but substantially reduced after cleaning. This p-doping is therefore likely to originate from residual PR [15,24,25].

**Figure 5.**
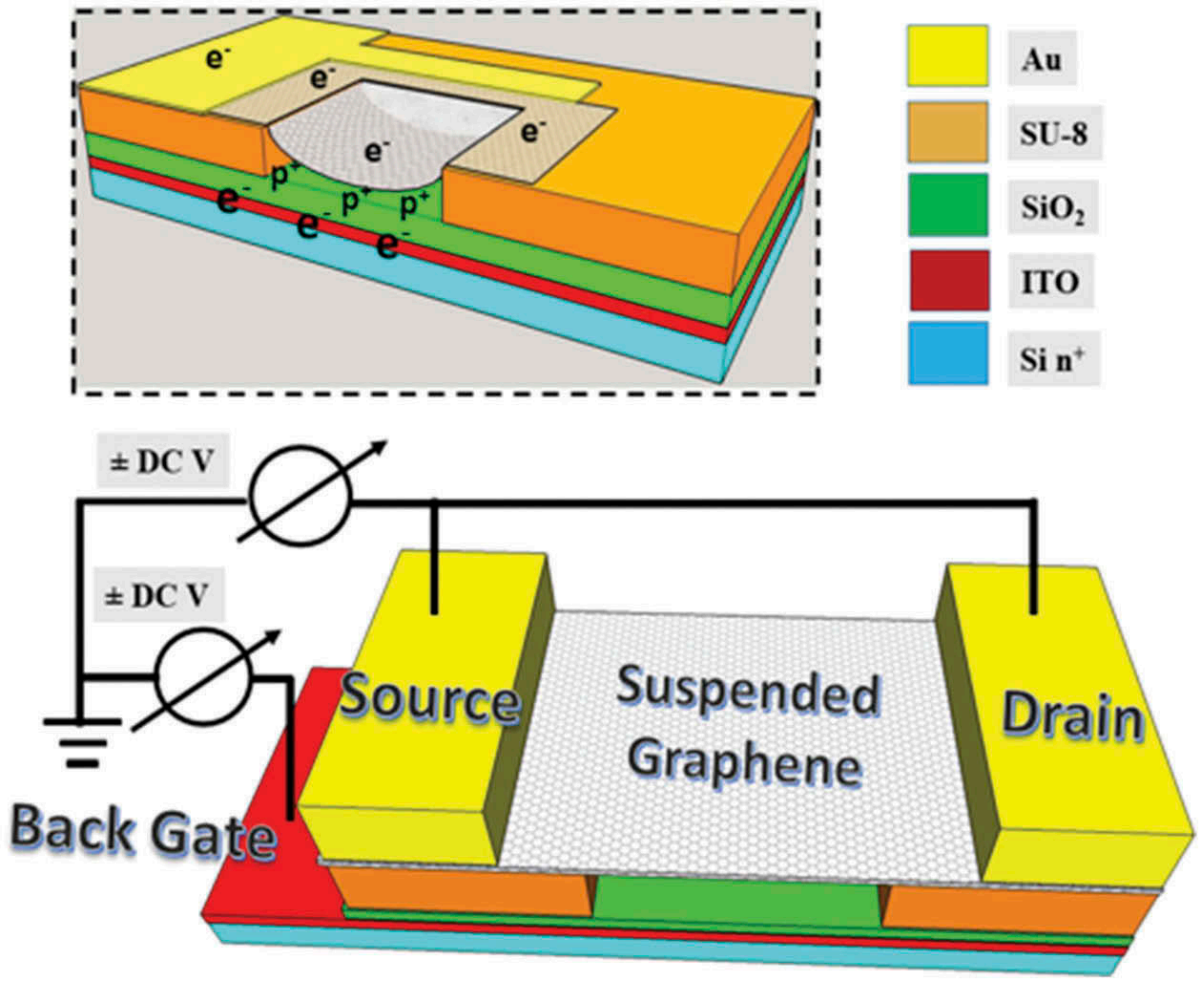
Device layout used to map the strain and Fermi energy as a function of applied biased. The source (S) and drain (D) are kept at the same voltage and a potential difference with the backgate voltage V_BG_ is established by varying V_S,D._

Moreover, once the residual PR is further removed through laser annealing (details of this procedure are given in SI, section 2), the RS data is different: e.g. the I(2D)/I(G) ratio exhibits variations across the surface and these can also be modulated by the application of a backgate bias voltage from −10 V to −30 V, as shown in Figure 4(c). Figure 5 depicts a 3D cartoon for the biasing configuration. This configuration is similar to a capacitor, with positive and negative plates controlling charges in between. The potential difference produces a systematic redshift of the energy peaks. Furthermore, the doping level is now very low, much less than 1 × 10^12^ cm^−2^ [54]. This rules out that doping is responsible for the local variations observed and therefore these must be due to other causes, either defects or strain. Since the I(2D)/I(G) ratio is low and the I(2D)/I(G) ratio is mostly close to 1, defects/non-uniformity of graphene sheet can be ruled out, and strain is the major reason behind the local variations observed.

Local variation in the RS data and resulting local strain can be associated with the existence of static ripples on the graphene surface, which are limited and constrained while the membranes are supported by a thin PR layer (associated with non-zero doping), but which become more pronounced and also tunable by an applied bias via the backgate, once the supporting action of the PR is removed (associated with zero doping). Already at 0 bias, such ripples result in mechanical deformation of the membranes, evidenced by substantial increases in the FWHM of the G bands (a well-known result of the splitting of the degeneracy of the G mode subbands) compared to a membrane with PR support. Some limited tunability can be further attained by applying a bias up to −30 V to the backgate with respect to the top contacts. Increasing the negative voltage reduces the FWHM, therefore one can conclude that either the strain decreases, changes in the Fermi energy are being triggered, or both. The apparent effect of mechanical deformation, i.e. strain, can, in fact, be linked to changes in the Fermi level of the graphene surface. According to Mohiuddin et al. [33] changes in the positions of the G and 2D modes can be directly related to strain:

(3) $$\Delta Pos{\left(G:2D\right)}_{\pm }^{s}=-Pos{\left(G:2D\right)}^{0}\gamma_{G:2D}\left(\varepsilon_{l}+\varepsilon_{t}\right)\left(1-\nu \right)\pm \frac{1}{2}Pos{\left(G:2D\right)}^{0}\beta_{G:2D}\left(\varepsilon_{l}-\varepsilon_{t}\right)\left(1+\nu \right),$$

where $\varepsilon_{l}$ and $\varepsilon_{t}$ are the longitudinal and transverse component of the strain. $\beta_{G:2D}$ the shear deformation potential. The experimental and theoretical value of the shear deformation potential is 0.99 [33]. The quantities $\left(\varepsilon_{l}-\varepsilon_{t}\right)$ and $\left(\varepsilon_{l}+\varepsilon_{t}\right)$ are the shear and hydrostatic components of the applied strain, respectively. The quantity $\gamma_{G:2D}$ is the Grüneisen parameter. The value of $\gamma_{G:2D}$ for the G (2D) mode is 1.99 [33] (1.24 [55]). The quantity ν is the Poisson’s ratio, which ranges from 0.33 for a good contact between graphene and its substrate to 0.13 for suspended graphene based on theoretical calculation [33]. In our study of suspended graphene, we selected 0.16 [56], which is the experimental value of graphite.

**Figure 6.**
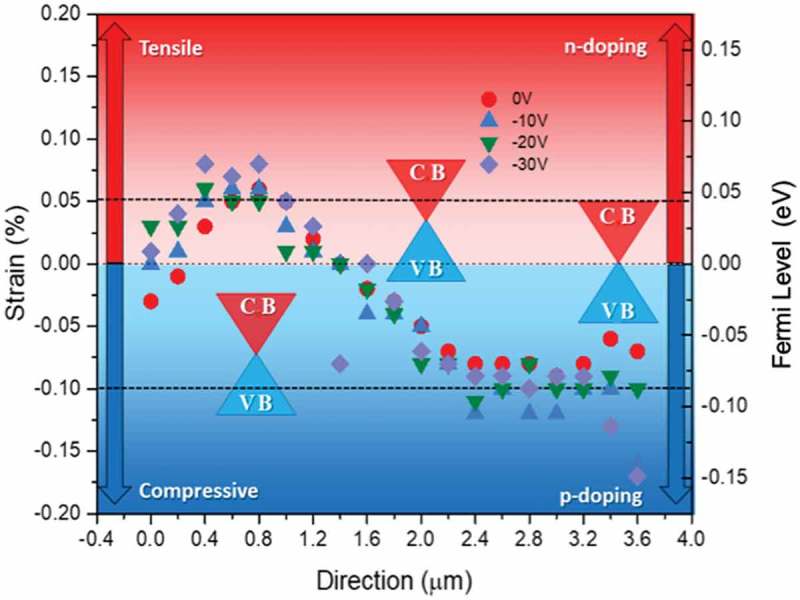
Strain and associated E_F_ variations across a single suspended graphene film. Both positive and negative values of the strain (tensile and compressive) are found and the Fermi level can shift positively/negatively giving rise to excess n/p carriers, forming charge puddles in the adjacent area at 0 V gate biasing. E_F_ tuning is also observed whilst varying the gate biasing.

Figure 6 depicts strain and associated Fermi level variations across a single suspended graphene film. Using Equation 3 to obtain the (uniaxial) strain, it can be seen how strain can vary from tensile to compressive in adjacent areas. Using Equation 1 the Fermi level can be also mapped, noticing that it appears to be almost directly proportional to the amount of identified strain, a result of the relative similarity of Equations 1 and 3.

It can be seen, from Figure 6, that at some locations on the SusG surface, the strain is zero, with E_F_ pinned at the Dirac point, i.e 0 eV. However, other sites have either compressive or tensile strain (blue and red areas), leading to a lower or higher Fermi level up to +70 meV and −110 meV with tensile and compressive strain, respectively. The Fermi level can shift positively/negatively giving rise to excess n/p carriers, therefore likely forming charge puddles in the adjacent area even at 0 V gate biasing.

These variations of the E_F_ can be further tuned by operating at different backgate voltages. Upon gate biasing, the electrostatic force will pull and stretch the surface of graphene thereby modifying the amount of strain and hence E_F,_ as shown in Figure 6 (more details about the distribution of strain in suspended graphene are given in SI, section 3).

**Figure 7.**
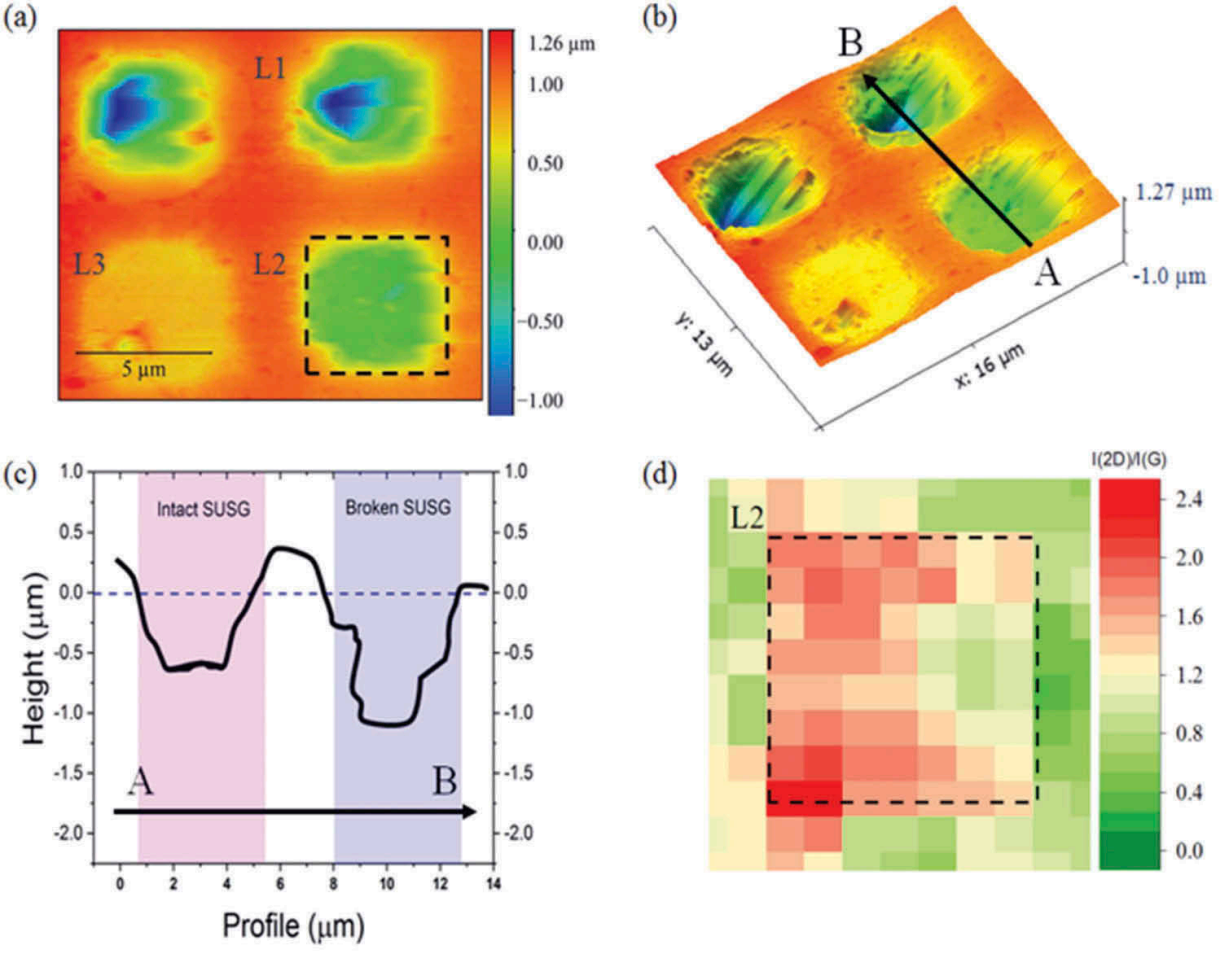
The morphology of sample #4 studied using AFM, SEM and Raman spectroscopy. (a) Selected locations: L1, broken SusG; L2, intact SusG and; L3, intact SusG with thicker residual PR. (b) 3D AFM micrograph. (c) Depth profiles: if we take the horizontal dashed line as the reference height for supported graphene, the depth of suspended graphene L2, is 600 nm compared to 2 μm for the broken SusG cavity, L1. (d) The intensity ratio I(2D)/I(G) confirms that the residual PR contamination on the surface of the SusG at L2 is suitably low compared to L3.

To show this, sample #4 was used. The morphology of sample #4 is first studied using AFM and scanning electron microscopy (SEM), just like the other samples. Three locations were selected: L1, broken SusG; L2, intact SusG and; L3, intact SusG with thicker residual PR (Figure 7(a)). Figure 7(b) depicts a 3D view of the image in Figure 7(a). If the horizontal dashed line (Figure 7(c)) is taken as the reference height for SupG, the depth of SusG sheet, L2, is 600 nm compared to 1.1 µm for the broken SusG cavity, L1, i.e there is a 400 nm separation between the SusG sheet and the base insulator layer on top of the backgate. This results in an increased electrostatic potential for the same voltage between the two samples. The intensity ratio I(2D)/I(G) (Figure 7(d)) confirms that the residual PR contamination on the surface of the SusG at L2 is suitably low compared to L3. To elucidate this, measurements related to sample #4, are obtained with a backgated configuration where the n-type silicon substrate was connected directly to a voltage source kept fixed at –10 V.

**Figure 8.**
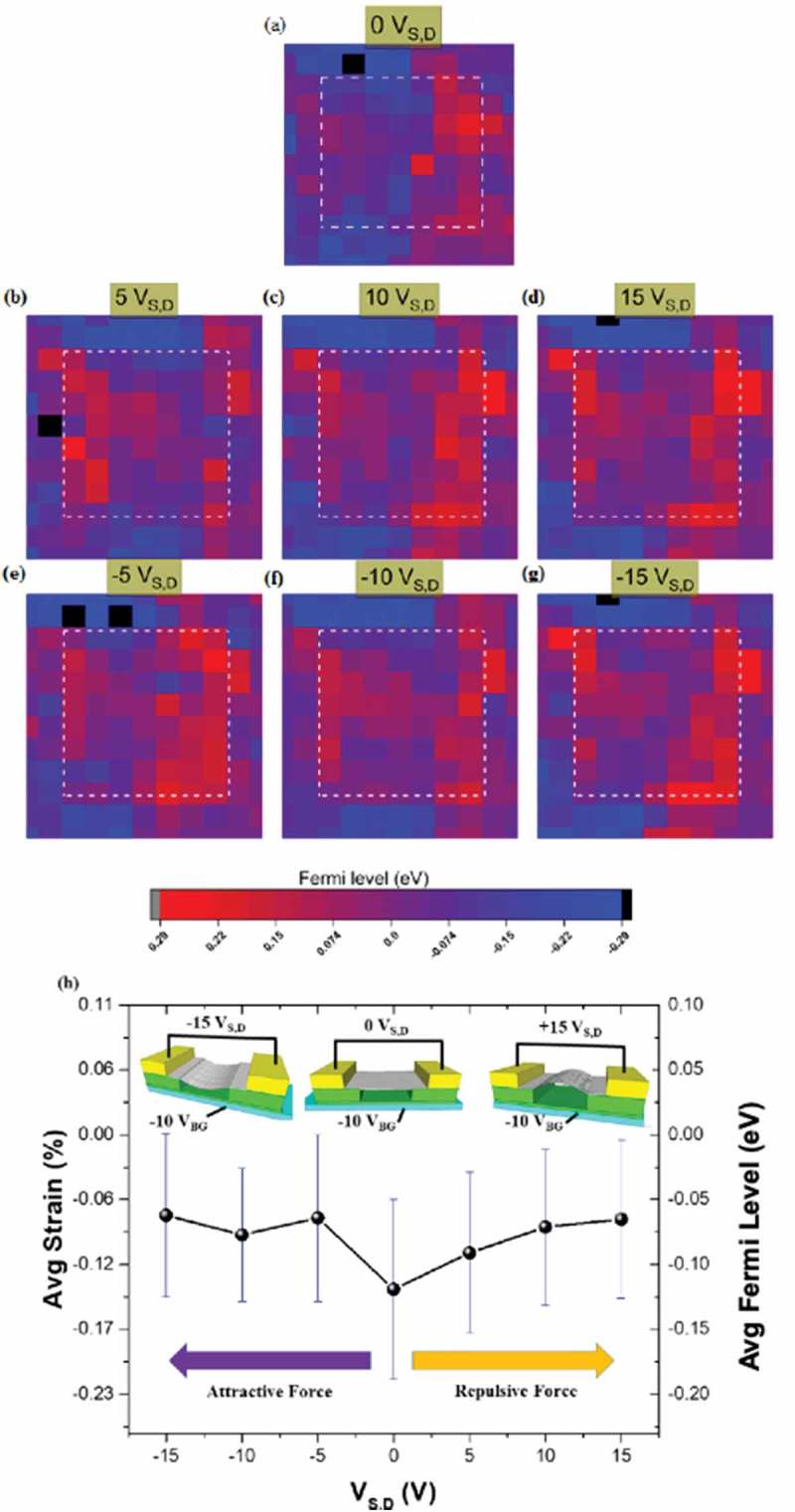
Sample #4: E_F_ maps under a fixed backgate voltage of −10 V, with (a) V_S,D_ = 0, and (b) to (g) as a function of applied V_S,D_ between −15 V and +15 V. The E_F_ oscillates between slightly positive and slightly negative values, corresponding to local tensile and negative strain. (h) The average value of the Fermi level over the SusG, showing negative (compressive strain). As V_S,D_ is varied, the Fermi level is reduced, irrespective of the polarity of V_S,D._

The source and drain contacts are instead kept at the same voltage V_S,D_, which was varied from −15 V to +15 V. The map in Figure 8(a) corresponds to the case of V_S,D_ equal to 0. In such case we notice how the E_F_ oscillates between slightly positive and slightly negative values, corresponding to local tensile and negative strain. The maps in Figure 8(b-g) show how changing V_S,D_ to a positive or negative non zero value forces changes in the Fermi level. Worth pointing out that as a dielectric covers the backgate contact, then the charges (positive) that appear on the surface of the dielectric are opposite to those of the backgate contact (negative). The average value of the Fermi level over the SusG (Figure 8(h)), is negative (compressive strain), which is perhaps surprising. As V_S,D_ is varied, a significant change in the Fermi level is noticed. It also appears that though changes of the local Fermi level depend on the applied V_s_, the average Fermi level value tends to start saturating quickly already at ±5 V. No differences between applying positive and negative values of V_S,D_ are observed, which is intuitively to be expected, as the strain produced by repulsion and attraction between the area of SusG and the bottom of its cavity, should be the same. The E_F_ is always reduced from its average value at V_s_ = 0. This is consistent with the proposition that both attraction and repulsion add a tensile component to the strain already present, lowering the amount of average compressive strain present on the suspended graphene area. Given the strain v Fermi level relationship outlined earlier, we can conclude that a tensile strain of up to 0.06% has been introduced by the external voltage. This demonstrates that dynamic tuning of the E_F_ is possible in a limited range of 0 to 50 meV.

Lastly, we need to discuss the macroscopic parameters such as mobility, conductivity (or resistivity), and why it is very difficult to directly extract such parameters for SusG. The simple reason is that much of the electrical behavior of the devices in this paper is dominated by SupG rather than SusG, as the membranes occupy a limited surface area in between the metal contacts (typically around 30% or less). This means that, e.g., the series resistance between SusG and SupG is always dominated by that of SupG, and any, e.g., increase in mobility or carrier concentration (or conductivity) becomes undetectable. Therefore, a completely different device layout, with limited SupG compared to SusG would be required to extract such parameters quantitively, which is the reason such values cannot be given in this work.

## Conclusions

Areas of suspended graphene have been obtained by transferring a CVD graphene film over cavities in either SU-8 or SiO_2_. We have studied a number of specimens and showed that large areas of up to 160 µm^2^ can be fabricated as functional backgated devices. There are significant hurdles in suspending arbitrarily large sizes, as the larger the area of suspended graphene, the deeper the cavity needs to be, to avoid the membrane sinking and touching the bottom of the cavity. We observed that static ripples are created on the suspended graphene and that such ripples introduce both tensile and compressive strain, simultaneously.

We have analyzed variations in the Fermi level in the suspended graphene areas, as a function of bias of the top contacts while having fixed the voltage of the backgate. We identify that the areas of suspended graphene mechanically react to being attracted/repelled to/from the backgate electrode. This mechanical deformation adds tensile strain to the film, and the Fermi level becomes less negative. We estimated that the tensile strain introduced in this way results in a change of Fermi energy of up to 50 meV, an appreciably large tuning. This effect can be exploited to detect different types of adsorbates sitting on the graphene surface. When the graphene is exposed to adsorbates, these will typically make a chemical bond with the surface. Our results show that strain and Fermi level can be effectively controlled by external voltage. Therefore, we postulate that: firstly, a voltage controllable shift in the Fermi level could be exploited to increase the adsorbate binding strength with the graphene surface; secondly, unlike supported graphene, suspended graphene will likely exhibit a substantial localized strain field when chemically bound to an absorbate. Such strain fields can in turn shift the Fermi level upwards or downwards relative to the Dirac point. These suggest that suspended graphene can form the basis of an electronic sensor with increased sensitivity.
