# Supplementary material for: Dynamic modulation of the Fermi energy in suspended graphene backgated devices
Source: Sci Technol Adv Mater. 2019 Jun 3;20(1):568–79. doi: 10.1080/14686996.2019.1612710 (PMC6567091; doi:10.1080/14686996.2019.1612710)
Supplement: Supplemental Material [file TSTA_A_1612710_SM7947.pdf]

SUPPORTING INFORMATION:

Dynamic modulation of the Fermi energy in suspended graphene backgated devices

Omar M Dawood[1,2,3], Rakesh Kumar Gupta[1,4], Umberto Monteverde[1], Faisal H. Alqahtani[1,5], Hong-Yeol Kim[1], James Sexton[1], Robert J Young[2,6], Mohamed Missous[1], Max A Migliorato[1]

[1]School of Electrical and Electronic Engineering, University of Manchester, United Kingdom

[2]School of Materials, University of Manchester, United Kingdom

[3]Department of Physics, College of Education for Pure Science, University of Anbar, Anbar, Iraq

[4]Manchester Institute of Biotechnology, University of Manchester, United Kingdom

[5]King Khalid University, Abha, Saudi Arabia

[6]National Graphene Institute, University of Manchester, United Kingdom

1. SEM/AFM/Raman; settings and systems used

We used an FEI Quanta as a multipurpose, high-performance tool to accommodate the imaging requirements for SEM. The following parameters were used for the Quanta 200 system to obtain accurate and clear information about the graphene surface:

1- High vacuum mode under pressure of 0.38 Torr.

2- Beam energy of 20 kV.

3- 10 mm working distance.

4- A spot size of 3 µm.

5- Secondary electron detector.

We have used two AFM systems so as to map the topography of our graphene samples. Topography images were captured using a commercial Dimension 3100 and an Asylum Instruments/Oxford Instruments MFP3D. AFM measurements were done using tapping mode in air under ambient conditions, with a scan speed of 0.5 line/second and a pixel resolution of 512 points/line. An AFM probe (SCM-PIT-V2, Bruker), was used in measurements with the following specifications:

I. Cantilever thickness, 2.80 µm.
II. Cantilever geometry, Rectangular.
III. Tip coating, PtIr.
IV. Tip Radius, 25 nm.
V. Resonant Frequency, 75 kHz.
VI. Spring Constant, 3.0 N/m.

In this project, we used the LabRAM (Horiba) Raman system to collect the Raman spectra of graphene. This system is equipped with an Ar+ (488 nm), associated with an optical microscope. For the LabRAM (Horiba) Raman, we used two Olympus objective lenses depending on the experiment and the sample. The first one is a Long Working Distance (LWD) objective lens, 50X magnification, 0.50 numerical aperture, 10.6 mm working distance, and a 2 µm diameter of the laser spot, which can provide a wide space between the lens surface and the graphene surface. The second one is a Short Working Distance (SWD) objective lens, 100X magnification, 0.90 numerical aperture, 0.21 mm working distance, and 0.7 µm the diameter of the laser spot, which can give us more accurate information about graphene. After we have done a series of tests, the laser power was maintained on the sample of about 1 mW/cm^2 so as to avoid damaging the sample due to heating effects. We can easily observe the effect of increasing the laser power by the background radiation, which can be emitted in the form of infrared radiation when the sample surface is hot. Different exposure and accumulation times, as well as extended and fixed mode, were selected depending on the nature of the sample and the results required. A 600 gr/mm grating, which defines the number of grooves per millimeter, was used to obtain Raman spectra with sufficient spectral resolution.

To be able to supply different polarity of voltages to our chips during Raman measurements, a programmable (Tektronix PWS4000) DC power supply, with a maximum output DC power supply of ± 60 V, was used. In addition, another power DC supply was used to operate the three terminals device during Raman measurements.

2. Graphene cleaning methods

1. 2.1 Acetone fog

The first technique is known as a chemical cleaning method using one type of common solvent. Acetone was used as a solvent, which is widely used to clean polymer layers from the surfaces of graphene. Acetone directly in contact with suspended graphene resulted invariably in tearing of the membrane. Therefore, keeping a specific distance between graphene and the acetone surface together with heating the acetone to a temperature close to its evaporation ~ 54 °C will reduce its surface tension to a degree that ensures that the polymer layers are dissolved and that graphene is not destroyed at the same time.

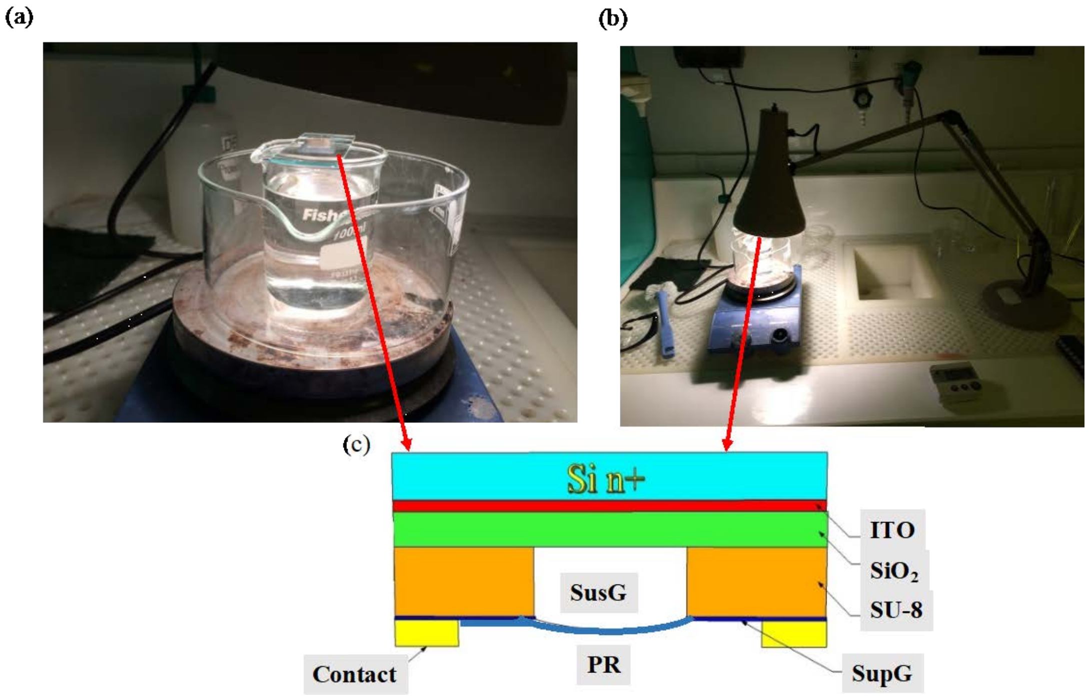

Figure S 1: Acetone Fog technique for cleaning suspended graphene using (a) a hot plate with two beakers of different sizes and (b) angle-poise lamps to provide a high intensity of the light. (c) Sample layout when positioned upside down.

Figure S 1 (a) shows the equipment used to clean suspended graphene from PR layer (Figure S 1 (c)). At first, the sample is fixed to a glass slide in a particular position using two pieces of the magnet. Then in order to heat the acetone, a hot plate was used as well as two beakers of different sizes. The small beaker is used to keep the acetone inside it at a high temperature while the other baker is working to ensure that the acetone vapour does not condense on the hot plate, leading to a potential hazard. When acetone vapour reaches the surface of the polymer, the temperature of the acetone will drop, leading to a change in the gas-to-liquid phase. As a result, there will be a high probability that graphene will be destroyed due to an increased surface tension of acetone. To avoid such a problem, we used an adjustable form of angle-poise lamps to produce a high intensity of the light supplied by its lamp to heat up the sample to high temperatures throughout the cleaning process, as shown in Figure S 1 (b).

2. *2.2 LASER irradiation*

It was observed in many of the samples examined after completion of the previous two processes, the (PR) layer was not completely removed, and some of the large-scale suspended graphene was broken. Hence, a laser technique was used as an alternative and effective solution [1],[2]. The removal of polymers is carried out by a laser cleaning process using specific conditions to ensure the cleanliness of graphene and also not to produce defects.

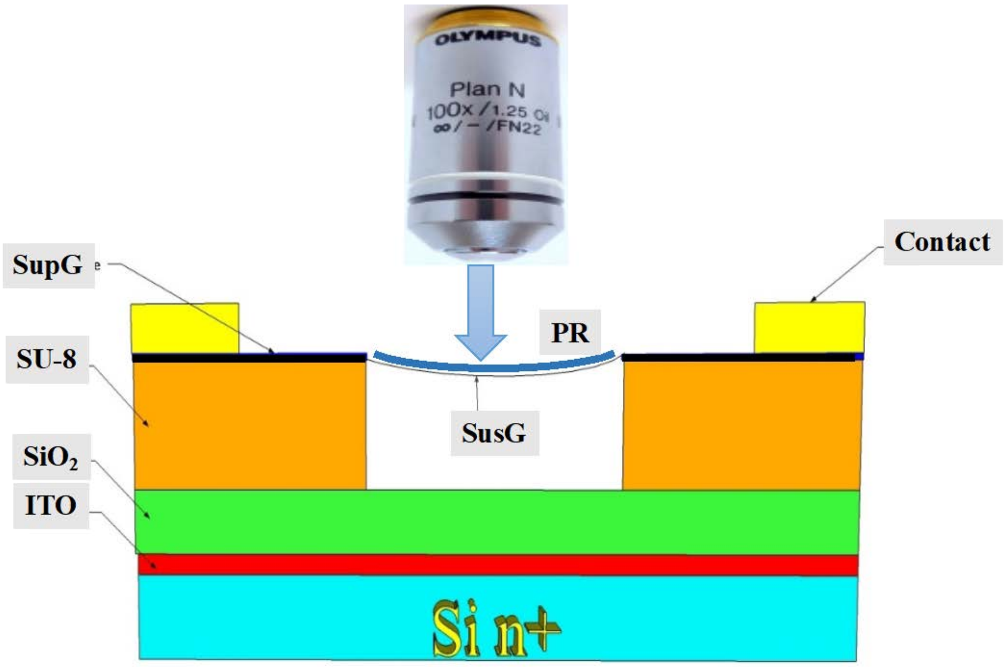

Figure S 2: LASER irradiation method for cleaning the suspended graphene

We used a visible laser of 488 nm with a 100X (short working distance) lens that is equipped with a LabRAM HORIBA Raman system, as shown in Figure S 2. The average value of laser power that touches the (PR) layer was carefully calibrated and measured using an optical power meter. The power ranges from 0.013 to 0.8 mW. These amounts of laser power are considered to be satisfactory to ensure an increase in the polymer etching rate. The exposure time of was 15 seconds with two accumulations. The laser beam is positioned on specific locations of graphene surface using a piezoelectric x-y stage. Raman measurements were used after eache single cleaning process to check whether the PR layer was eliminated entirely or not and also to guarantee no defects were introduced in the graphene.

3. Raman spectroscopy results

Raman spectrum of the 5x5 μm^2 size of suspended graphene was acquired. The objective was to know how much power and time we have to use for future cleaning tests. The cleaning process lead to improvement identifiable through the Raman spectrum when the elimination of PR particles began to take place by the heat that was provided by the incident LASER power.

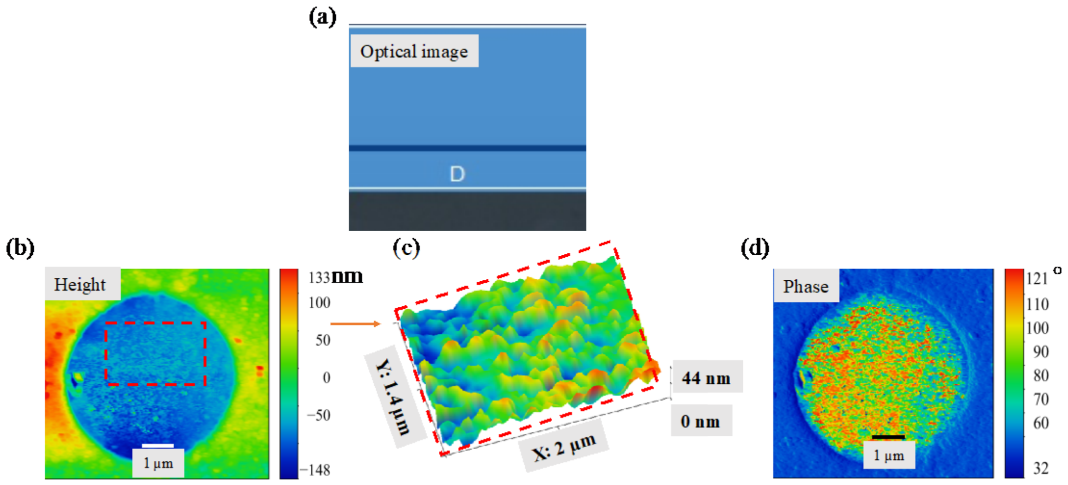

Figure S 3: The results of cleaning the surface of suspended graphene by Laser method as can be shown by (a) optical images and (b) 2D AFM height image and the (c) 3D inset image shows the topographical feature of graphene surface after removing the PR layer and (d) the 2D phase AFM image of the suspended graphene surface. The scale bar is 1 µm.

To be able to monitor precisely the amount and type of doping concentrations in graphene, Raman spectroscopy is the most desirable tool. The ratio I(2D)/I(G), Pos(G;2D) and FWHM(G;2D) are the most prominent parameters used to detect the reliability of the annealing experiment. Two conditions have to be taken into account for performing such a standard cleaning method: the defect density expressed by the ratio I(2D)/I(G) has to be minimized and the cleaning should be irreversible, i.e. no doping charges can again affect the graphene.

Figure S 4 (a) shows the Raman spectra of graphene with the two main modes, G and 2D, at each five heating cycles, at specific incident LASER power. At first heating cycle, 0.013 mW, the background of the spectrum of graphene is not flat reflecting the effect of PR residues on SusG sheet, which can be detected at the wide peak that starts at 1400 cm^{-1} up to 1700 cm^{-1}. The peak of the G mode, therefore, has to be extracted by performing a proper fitting process. We can observe the smoothest of the background at the third heating cycle, 0.8 mW, indicating the reduction of the amount of PR and the peak around the G mode starts to become narrower. We repeated the last power, 0.8 mW, to be sure no more PR remains. The last heating cycle, 0.013, we can notice clearly the spectrum of graphene is similar to a pristine monolayer graphene.

Now we need to study the stability of the laser method and the effect of increased temperature on the G and 2D mode of graphene. Figure S 4 (b), shows the intensity ratio I(2D)/(G) is increased when the heating temperature rises, confirming the reduction level of doping concentrations. The red-shift of the Pos(G;2D), Figure S 4 (c), is due to the increased temperature of graphene which strectches the carbon-carbon bonds. In addition, heating effects can also be observed by the increased broadening of the FWHM(G;2D). At the final heating cycle, 0.013 mW, the values of the I(2D)/(G) Pos(G;2D) and

FWHM(G;2D) refer to an uncontaminated monolayer graphene but with a minor level of defects introduced in graphene after cleaning.

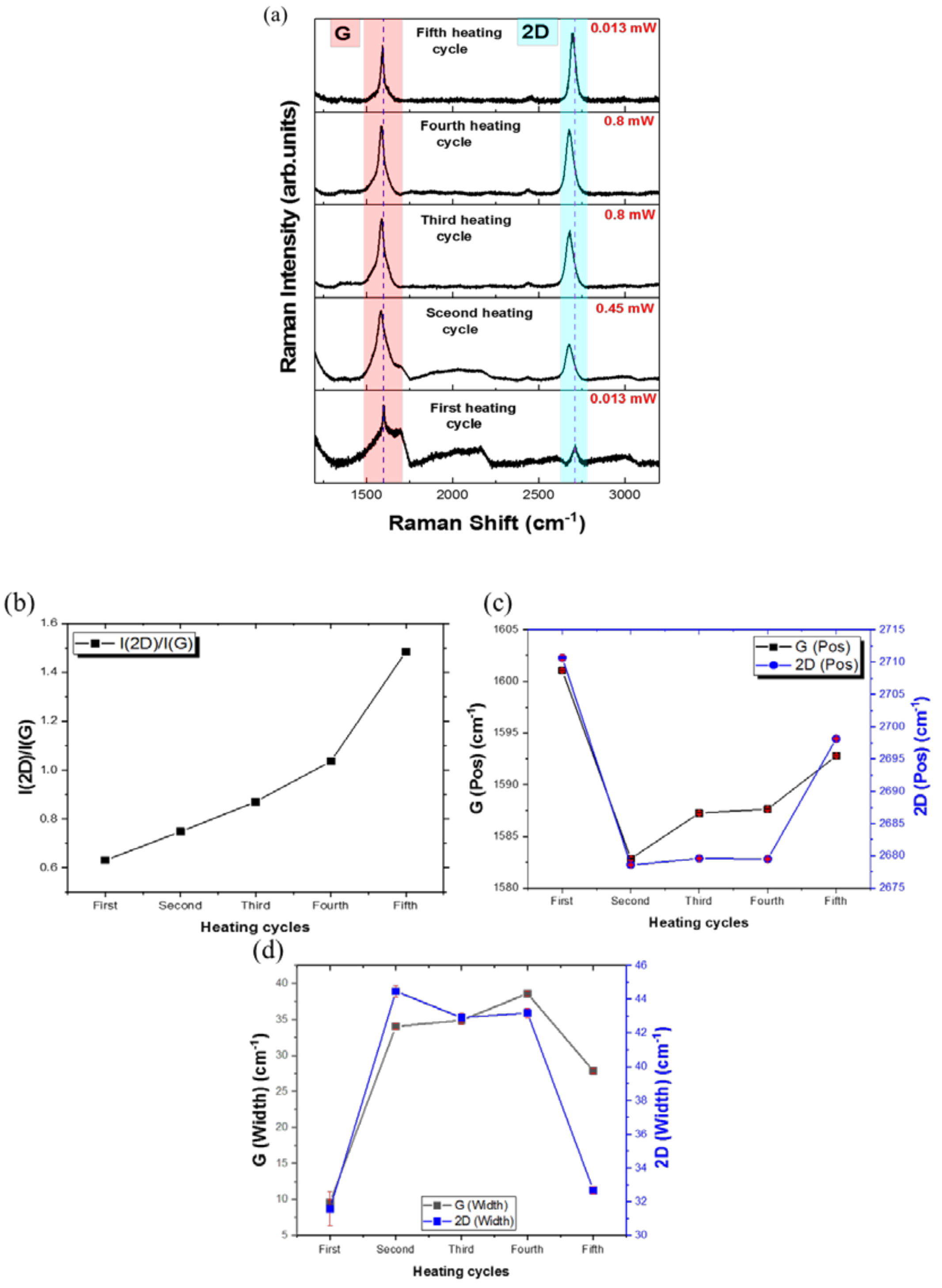

Figure S 4: (a) Raman spectrum of graphene after performing five heating cycles at a specific incident LASER power as well as the (b) intensity ratio I(2D)/I(G), the (c) peak position shifts of the G and 2D mode and the (d) the full width at half maximum of the G and 2D mode before and after cleaning the surface of suspended graphene.

The experiment was repeated using the same laser power and exposure time on a rectangular cavity of 10×3 μm^2 in order to check the consistency of the laser cleaning mothed.

We chose 19 selected points with an explicit distance of 200 nm for Raman cleaning test, before and after cleaning PR. Raman features of these given points are discussed in details in the main body of the paper (section 5). A summary of Raman Spectroscopy results confirms the elimination of PR on top of the SusG sheet.

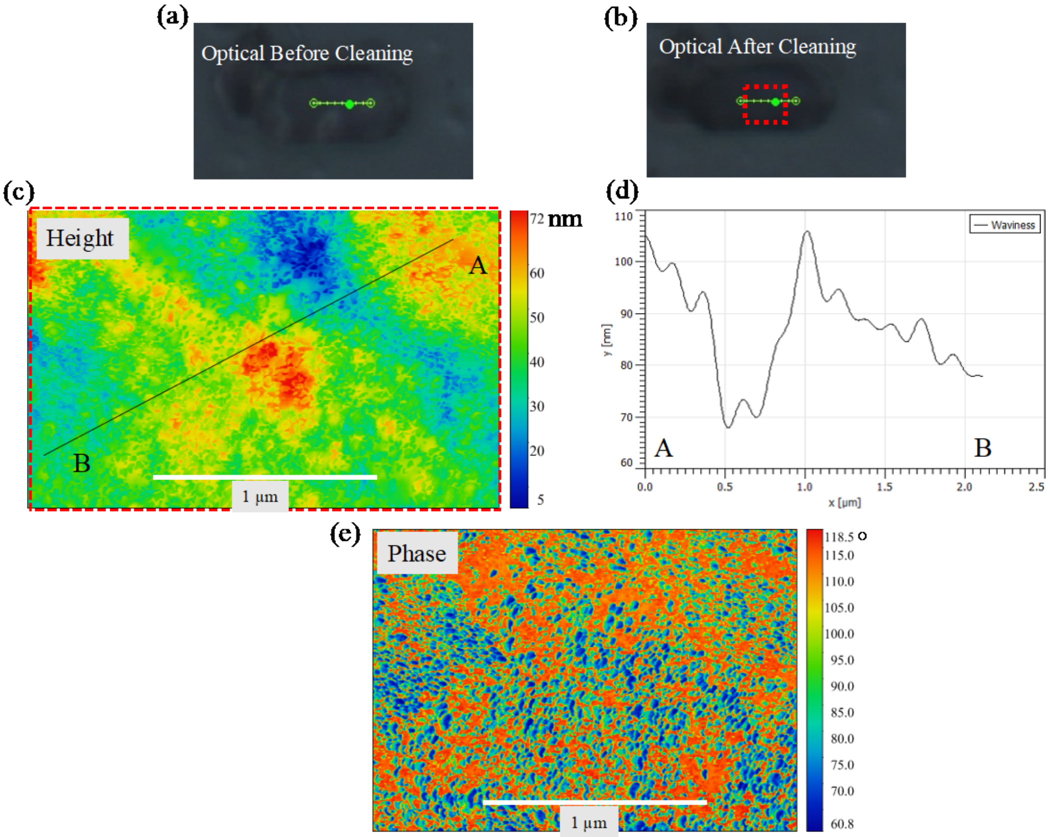

Figure S 5: Cleaning the surface of suspended graphene by laser annealing of a rectangular cavity of 10×3 μm. (a) before and (b) after cleaning the PR. In addition, the (c) 2D AFM height image and the (d) 2D inset image shows the undulation of the graphene surface after removing the PR layer and (e) the 2D phase AFM image confirms the elimination of PR on the suspended graphene surface.

The optical images, presented in Figure S 5 ((a) and (b)), clearly show the removal of the PR layer by comparing the light contrast of the optical image before and after the cleaning process is completed.

We now turn to the morphology of suspended graphene, to study the effect of strain on tuning the Fermi level of clean SusG (more details are presented in the main body of the paper section 5). From the AFM height and inset image, Figure S 5 (c), we can observe the formation of ripples on suspended graphene Also, the AFM phase discriminates (Figure S 5 (d) between the surface of graphene (at a phase degree of around 120˚) from the PR surface (at lower phase of 60˚), similarly to the previous case.

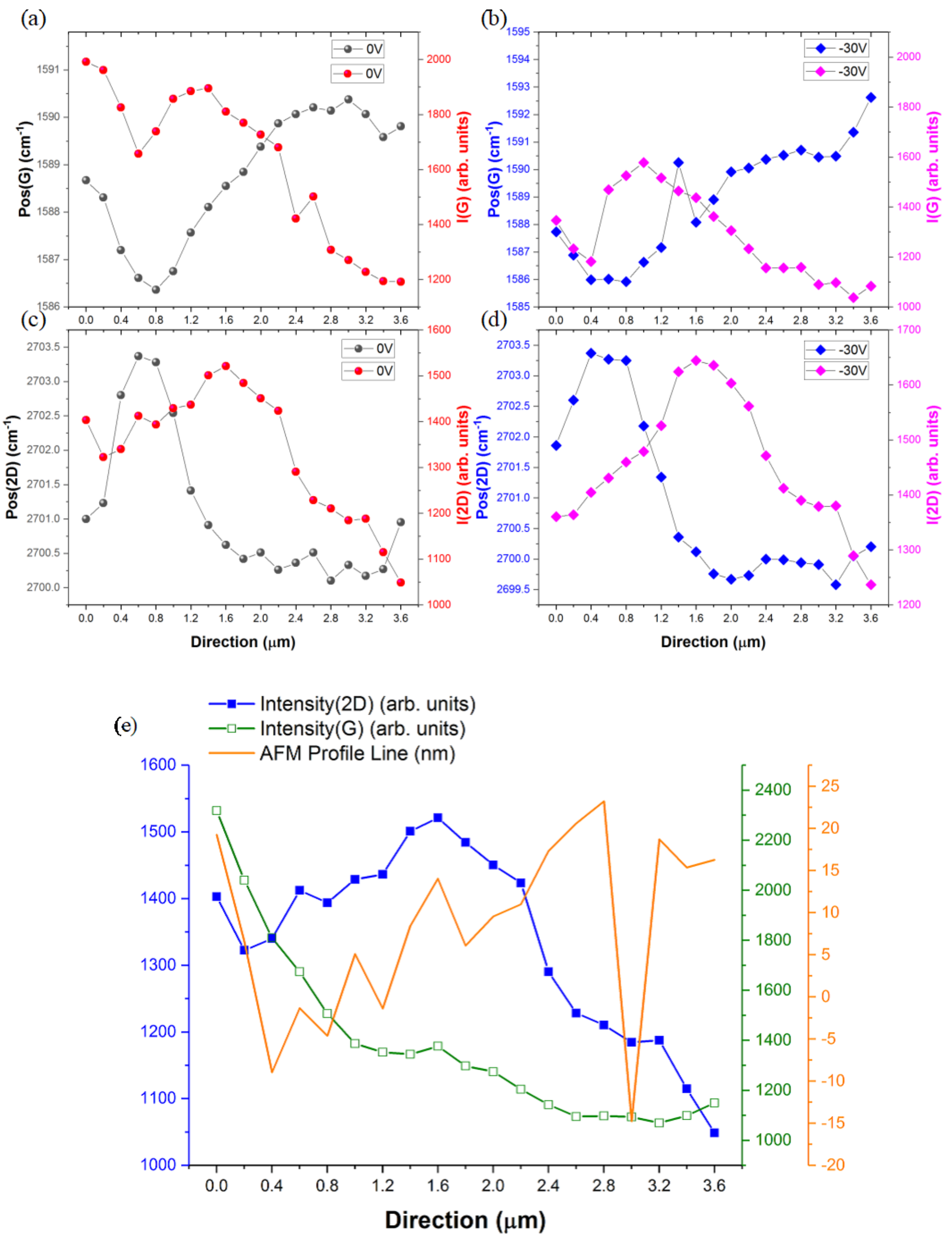

Figure S 6: Confirmation of the existence of ripples in suspended graphene sheet by the inverse relationship between the peak positions of the G mode with its intensities at (a) 0V and (b) -30 V bias voltages. The same inverse relationship of the peak positions of the 2D mode with its intensities is observed at (c) 0V and d) -30 V bias voltages. In addition, the (e) intensities of the G and 2D mode match the ripple features of the SusG sheet.

The existence of ripples in suspended graphene can be confirmed by the inverse relationship between the Pos(G) and Pos(2D), Figure S 6 ((a) and (b)), with their intensities, Figure 6 5 ((c) and (d)). The intensity of Raman modes follows the undulated surface of graphene's ripples. The observations are consistent with the observations of Polyzos *et al* [3]. Also, we can observe, in Figure S 6 (e), the equivalent result in terms of relationship between the AFM profile line with the intensity of the G and 2D mode.

4. SEM images of partially broken SusG

Figure S 7 shows that the cavity is not fully covered by the SusG sheet because some parts are broken as a result of increased pressure on the surface of the graphene after the hot acetone cleaning process. Van der Waals forces are responsible for SusG adhering to the cavity side walls. Some broken parts appear to still adhere to the sidewall of the cavity, hanging by a thread. The conclusion is that the condition for obtaining a large area of intact graphene sheet is that the force exerted by the weight of the liquid used to clean the surface of the graphene, which is pushing the membrane downwards, should not exceed the Van der Waals forces that instead push the membrane sideways.

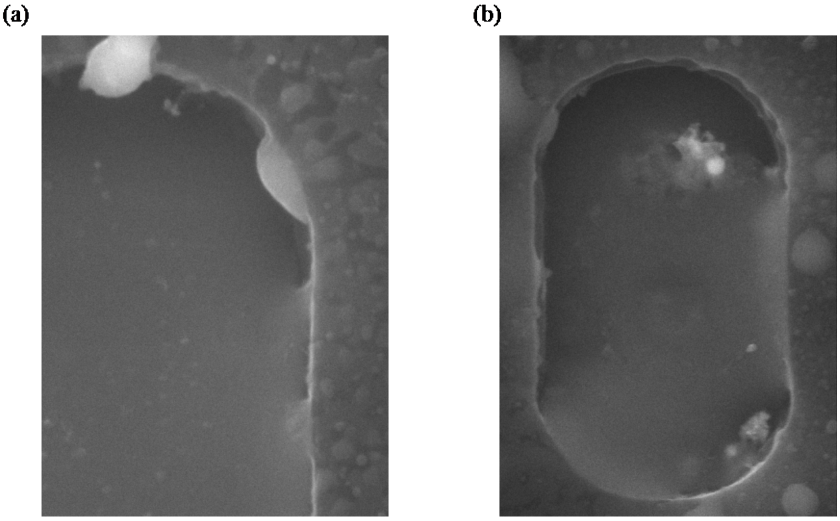

Figure S 7: The cavity in (a) and (b) shows parts of the broken suspeded graphene sheet attached the the side wall of the cavity.

5. Strain mapping of sample #4

We examined the Raman spectra of the suspended graphene sheet of sample #4 at 0 volt bias in order to confirm the presence of inherent static strain in SusG upon removing of PR layer after the cleaning process. The strain can be determined by analyzing the peak shifts of both Raman modes as well as the width of the FWHM(G). In addition, the width of the FWHM(G) can be used to test the doping concentration introduced in graphene.

In Figure S 8 ((a) and (b)), show that both the Pos(2D) and Pos(G) distributed within the SusG area are mostly red-shifted compared to the Pos(2D) and Pos(G) of the SupG area. The type and amount of static strain generated on the SusG area are distributed non-uniformly, confirming the strain is anisotropic. The existence of strain is also supported by the broadening of the FWHM(G) within the area of SusG, as shown in the mapping result of Figure S 8 (c), compared to the mapping distribution of the FWHM(G) of SupG area.

We now turn to examine the effect of applying different bias voltages on the suspended graphene sheet. This effect lies in increasing the constant stress value of graphene sheet due to the increased the physical

mobility of the graphene sheet. Applying different bias voltages between the graphene and backgate will result in the generation of the attractive and repulsive forces that give a cause of tension in graphene due to the increase or decrease of the bonds between the carbon atoms. This expansion (contraction) in the C-C bond will result in an increase in tensile (compressive) strain. The heterogeneous distribution of Pos(G) in Figure S 9 ((b) to (g)) confirms the existence both tensile and compressive strain on the SusG sheet.

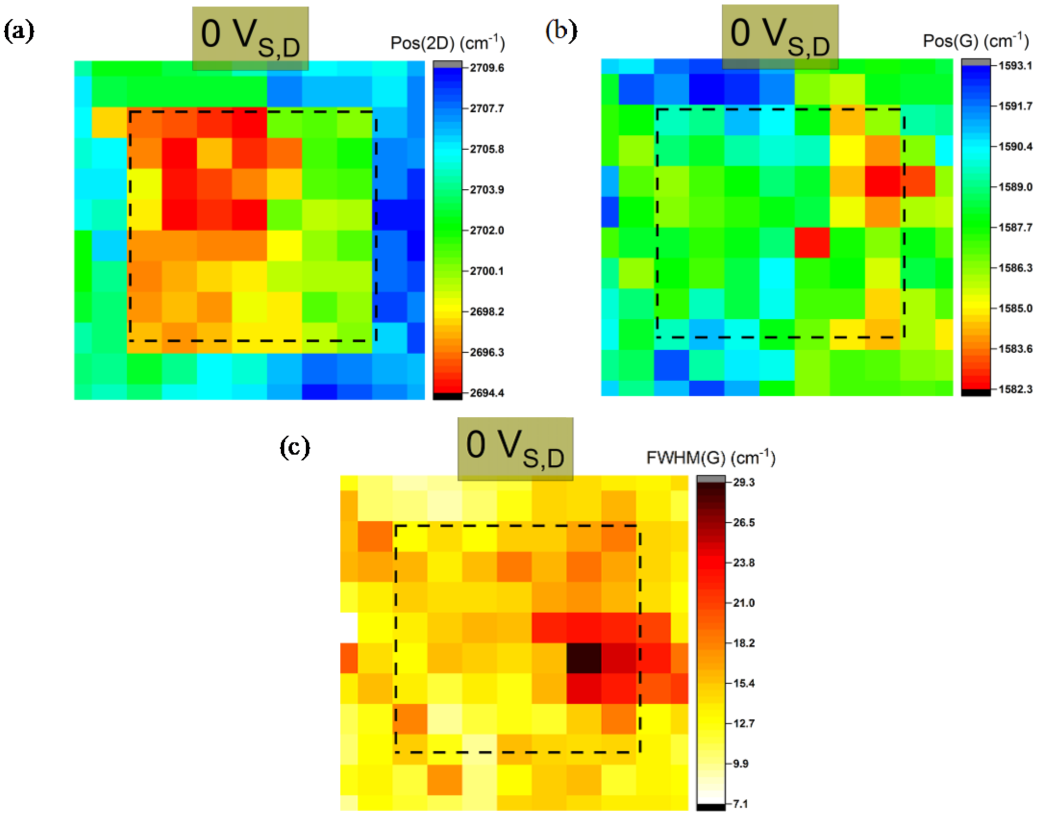

Figure S 8: Raman mapping results of sample #4 confirming the existence of static strain in a suspended graphene sheet by measuring the red-shifted peak positions of the (a) 2D and (b) G mode. The lower level of doping concentration and a larger amount of strain can be confirmed by observing the (c) FWHM(G) in the suspended graphene area.

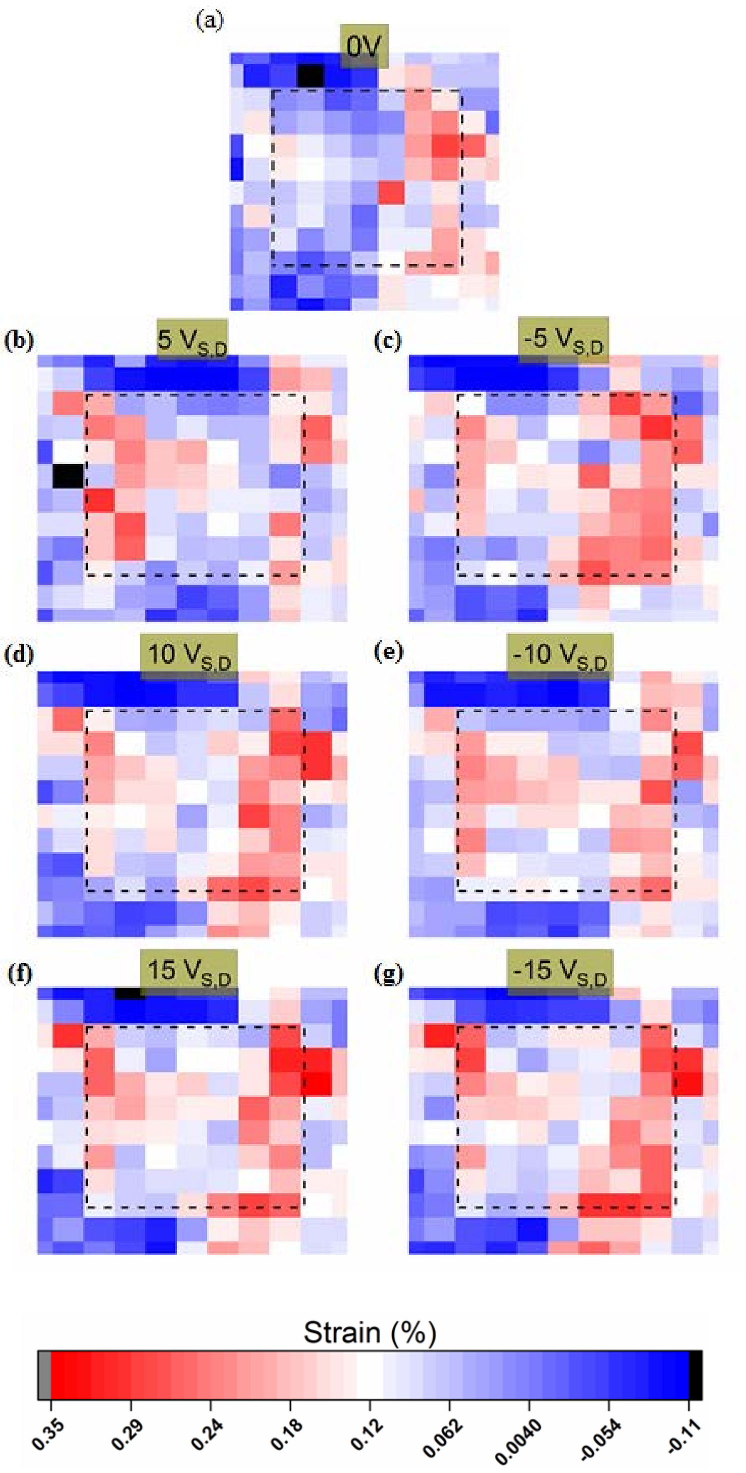

Figure S 9: (a) Distribution of strain within the area of SusG at 0V $V_{S,D}$ and $V_{BG.}$ From b) to g) the effect of different applied voltages between the sheet of suspended graphene and the backgate on increasing the amount and type of induced strain.

References

[1] J. Moser, A. Barreiro, A. Bachtold, Current-induced cleaning of graphene, Applied Physics Letters 91(16) (2007) 163513.

[2] K.I. Bolotin, K.J. Sikes, Z. Jiang, M. Klima, G. Fudenberg, J. Hone, P. Kim, H.L. Stormer, Ultrahigh electron mobility in suspended graphene, Solid State Communications 146(9-10) (2008) 351-355.

[3] I. Polyzos, M. Bianchi, L. Rizzi, E.N. Koukaras, J. Parthenios, K. Papagelis, R. Sordan, C. Galiotis, Suspended monolayer graphene under true uniaxial deformation, Nanoscale 7(30) (2015) 13033-13042.
